# Effects of Intra-Articular Autologous Adipose Micrograft for the Treatment of Osteoarthritis in Dogs: A Prospective, Randomized, Controlled Study

**DOI:** 10.3390/ani12141844

**Published:** 2022-07-20

**Authors:** Riccardo Botto, Valentina Riccio, Livio Galosi, Giacomo Rossi, Silvia Vincenzetti, Adolfo Maria Tambella, Francesco De Francesco, Luca Pennasilico, Michele Riccio, Alberto Salvaggio, Sara Sassaroli, Angela Palumbo Piccionello

**Affiliations:** 1 School of Biosciences and Veterinary Medicine, University of Camerino, Via Circonvallazione 93/95, 62024 Matelica, Italy; riccardo.botto@unicam.it (R.B.); valentina.riccio.dvm@gmail.com (V.R.); livio.galosi@unicam.it (L.G.); giacomo.rossi@unicam.it (G.R.); silvia.vincenzetti@unicam.it (S.V.); adolfomaria.tambella@unicam.it (A.M.T.); luca.pennasilico@unicam.it (L.P.); alberto.salvaggio@icloud.com (A.S.); sara.sassaroli@studenti.unicam.it (S.S.); angela.palumbo@unicam.it (A.P.P.); 2 Department of Reconstructive Surgery and Hand Surgery, University Hospital (AOU Ospedali Riuniti di Ancona), Via Conca 71, 60123 Ancona, Italy; michele.riccio@ospedaliriuniti.marche.it

**Keywords:** Rigenera^®^, regenerative therapy, osteoarthritis, dog, microfragmented adipose tissue

## Abstract

**Simple Summary:**

Osteoarthritis (OA) is a very common musculoskeletal condition that affects dogs, as well as humans and other species, and causes pain, lameness, and disability. Since therapy is not always effective and is definitive only in some cases, OA is still a leading cause of euthanasia in dogs and a challenge for orthopedic surgeons. Recently, new therapeutic approaches, such as the use of autologous mesenchymal stem cells, have been developed. The purpose of this prospective, randomized, controlled, in vivo clinical study was to estimate the safety, feasibility, and efficacy of the intra-articular treatment of autologous microfragmented adipose tissue in dogs affected by spontaneous OA in comparison with a treatment with hyaluronic acid. Pain, lameness, the radiographic progression of OA, and synovial fluid inflammation were assessed. The results suggest that intra-articular injection of microfragmented autologous adipose tissue is safe, timesaving, cost-effective, minimally invasive, and can be easily done in one step. This treatment, compared with the hyaluronic acid treatment, showed better long-term pain control and an amelioration of synovial fluid quality, resulting in an improvement in joint function. This new treatment can be included among the effective therapies for OA. Additionally, the canine spontaneous OA model adopted in this study could play a key role in developing successful treatments for translational medicine.

**Abstract:**

The purpose of this study was to estimate the safety, feasibility, and efficacy of the intra-articular treatment of autologous microfragmented adipose tissue in dogs with spontaneous osteoarthritis (OA) in comparison with hyaluronic acid (HA), the standard intra-articular treatment. Specifically, it clinically evaluated pain and lameness, the radiographic progression of osteoarthritis, and synovial fluid inflammation. This was a prospective, single-center, parallel-group, randomized, controlled, in vivo clinical study. Participants (*n* = 40) received either a single intra-articular injection of microfragmented adipose tissue or a single intra-articular injection of HA (1:1). Clinical outcomes were determined using a specialistic clinician assessment obtained by the completion of a specific clinical form based on the Vesseur modified lameness classification system, a pain evaluation using the Visual Analogue Scale (VAS), the measurement of the range of motion (ROM) of the affected joint, limb circumference, and the owners’ score evaluation using the Canine Brief Pain Inventory (CBPI) for up to 6 months after the time of injection. Patients underwent a radiographic examination to establish the degree of OA in the affected joint, and synovial fluid samples were collected to assess the biochemical environment of the joint and evaluate and quantify the cellular population and the presence of three specific inflammation biomarkers for up to 60 days. The results of this study suggest that microfragmented autologous adipose tissue is safe and can effectively relieve pain and improve function in dogs with spontaneous articular OA. This one-step procedure is simple, timesaving, cost-effective, minimally invasive, and eliminates the need for complex and time-intensive cell culture processing. Furthermore, the clinical evidence and cytological results suggest better long-term pain control, resulting in an improvement in joint function, compared to HA treatment. The canine spontaneous OA model could play a key role in developing successful treatments for human medicine.

## 1. Introduction

Osteoarthritis (OA) is a chronic disease affecting joints [1], characterized by a progressive deterioration of articular cartilage, the formation of periarticular osteophytes, and inflammation of the synovial component, resulting in pain and a progressive loss of joint function [2]. The incidence of this pathology is high in canines as well as humans [3,4,5]. Canine OA is generally considered to bear a close resemblance to human OA with regard to anatomic similarity, disease heterogeneity, and progression [6]. For this reason, several researchers in human medicine have used dogs with spontaneous OA to study the disease, and thus, this species is ranked as the best animal model to adopt for research [7]. In consideration of the fact that OA is a multifactorial pathology and there is no definitive cure able to stop the progression of the pathology [5,8,9], in the last few decades, the scientific community has concentrated a growing degree of interest on the experimentation and study of alternative conservative therapies that may be able to heal patients with OA [10,11,12]. In particular, researchers in the field of regenerative medicine have focused on the therapeutic properties of mesenchymal stem cells (MSCs) to better understand the feasibility and efficacy of their use [13,14]. Although mesenchymal stem cells were originally isolated from bone marrow [15,16], similar populations have been reported in other tissues, such as human adipose tissue [17], umbilical cord blood [18,19,20,21], peripheral blood [22,23], connective tissues of the dermis, and skeletal muscle [24]. Due to the characteristic high concentration of stem cells, easy isolation, and its plastic properties, the scientific community has shown a particular interest in MSCs derived from adipose tissue, which are called adipose-derived stem cells (ASCs) [25,26,27,28,29,30,31]. Once processed, the adipose tissue’s aqueous fraction, consisting of ASCs, and its stromal vascular fraction (SVF) can be extracted. The SVF contains precursors of endothelial cells (EPCs), macrophages, smooth muscle cells, lymphocytes, pericytes, and preadipocytes [29]. The clinical efficacy of treatment of OA through SVF infiltration is linked to the SVF’s anti-inflammatory and immunoregulatory effects, alongside the regenerative capacity of ASCs [25]. MSCs are a promising candidate for cartilage regeneration due to their ability to differentiate towards cartilage and bone cells and secrete trophic factors with regenerative functions linked to their paracrine, antiapoptotic, and anti-inflammatory effects [30]. The use of SVF and ASCs as a valid alternative therapy for the treatment of OA has already been validated by in vitro studies that have demonstrated the presence of markers such as CD73, CD90, and CD106, which are necessary for cell differentiation into cartilage [31,32]. Moreover, in vivo studies have also reported interesting results, despite having their limits [33,34]. Over the past decade, research has brought new insights into the effects of ASCs, and new mechanical disintegration technologies promise rapid, ready-to- use stem cell collection under the principle of “minimum manipulation” [35,36,37,38].

With this study, we evaluated the safety, feasibility, and effectiveness of autologous microfragmented adipose tissue (MFAT) intra-articular injections, obtained with a system for the disaggregation of adipose tissue, for the treatment of spontaneous OA in dogs, comparing this treatment to that of using HA. The primary target of the study concerned the evaluation of the efficacy of MFAT expressed as a reduction in pain and degree of lameness and the persistence of clinical improvement in the short and long term. The secondary target was based on the evaluation of the regenerative effect of MFAT treatment in vivo, currently demonstrated by the authors in vitro only, expressed in terms of improvement in the degree of radiographic osteoarthritis and the quality of synovial fluid in the study evaluation times after T0.

## 2. Materials and Methods

### 2.1. Ethics Statement

This study was reviewed by the Animal Welfare Body of the University of Camerino and received formal institutional approval (protocol n. 1D580.18A) in accordance with national and European law.

### 2.2. Eligibility Criteria and Dogs’ Enrollment

Forty pet dogs affected by OA were referred by the Veterinary Teaching Hospital of the University of Camerino and enrolled in the study with their owners’ consent. Eligibility criteria included dogs aged 1 to 15 years, with no weight and sex restriction, belonging to the ASA 1–2 anesthetic risk class [38]; absent of comorbidity, pregnancy, or lactation; affected by lameness manifesting in one joint caused by OA; absent of OA in other joints of the same limb; belonging to radiographic grade OA between 1 and 4 (according to a modified Kellgren-Lawrence scale) [39]; and having not received anti-inflammatory drugs nor nutraceuticals in the 15 days prior to enrollment in the study. The entire cohort of patients who concluded the study had to follow a management therapy after receiving the treatment, limiting uncontrolled activities, favoring leashed walks, and avoiding nutraceuticals or anti-inflammatory drugs. The patients that required administered anti-inflammatories were excluded from the research. In addition, in order to avoid significant changes in body weight during the study period, all patients were subjected to a controlled diet, favoring the administration of rationed commercial food according to the indications given on the packaging.

### 2.3. Clinical Trial Procedures

The enrolled dogs were randomly divided into equal groups of 20 patients, a study group and a control group, which differed only by their received intra-articular therapies. Anamnestic data and the owners’ perception of pain, by means of the Canine Brief Pain Inventory (CBPI), were collected. Subsequently, a specialist clinician conducted a general and orthopedic specialist visit, filling out a clinical record. After the clinical assessment, a radiographic examination under anesthesia was performed to confirm and grade the OA. During sedation, the affected joint was trichotomized, and synovial fluid was collected by arthrocentesis for qualitative cytological examination. The residual synovial fluid was stored in a −80 °C freezer to quantify the concentration of synovial cytokines. Each patient followed the same anesthetic protocol for radiographic, intra-articular therapy, and synovial evaluation, reported in the proper paragraph. At time 0 (T0), the entire study population was treated with a single intra-articular injection, using different products according to the designated group. The clinical, radiographic, and synovial fluid assessments were performed at T0 and repeated after 30 (T1) and 60 (T2) days in both groups. After 180 days (T3), the cohort of patients was re-examined to collect data related to the perception of pain by the owner, through the Canine Brief Pain Inventory (CBPI), and they attended a subsequent final orthopedic specialist visit. A summary of the timing of therapeutic and evaluation procedures is shown in Table 1.

### 2.4. Anesthesiological Protocol

Patients were sedated with 3 μg/kg of dexmedetomidine and 0.2 mg/kg of methadone IM and then anesthetized by 2–3 mg/kg of propofol IV until tracheal intubation was achieved. Anesthesia was maintained with 1.2% isoflurane in oxygen for the whole procedure.

### 2.5. Control Group Therapy (Hyaluronic Group)

At T0, dogs randomly enrolled in this group received a single intra-articular injection of high molecular weight (650 kDa) hyaluronic acid (20 mg/2 mL Hyalgan^®^, Fidia Farmaceutici S.p.A., Abano Terme, Italy). The dosage of the product was between 1 and 2 mL: this was optimized according to the affected joint and the patient’s constitution so as not to overextend the joint capsule, causing pain in the days following the treatment. The procedure was performed using good hygiene and aseptic practices.

### 2.6. Study Group Therapy (MFAT Group)

**Figure 1:**
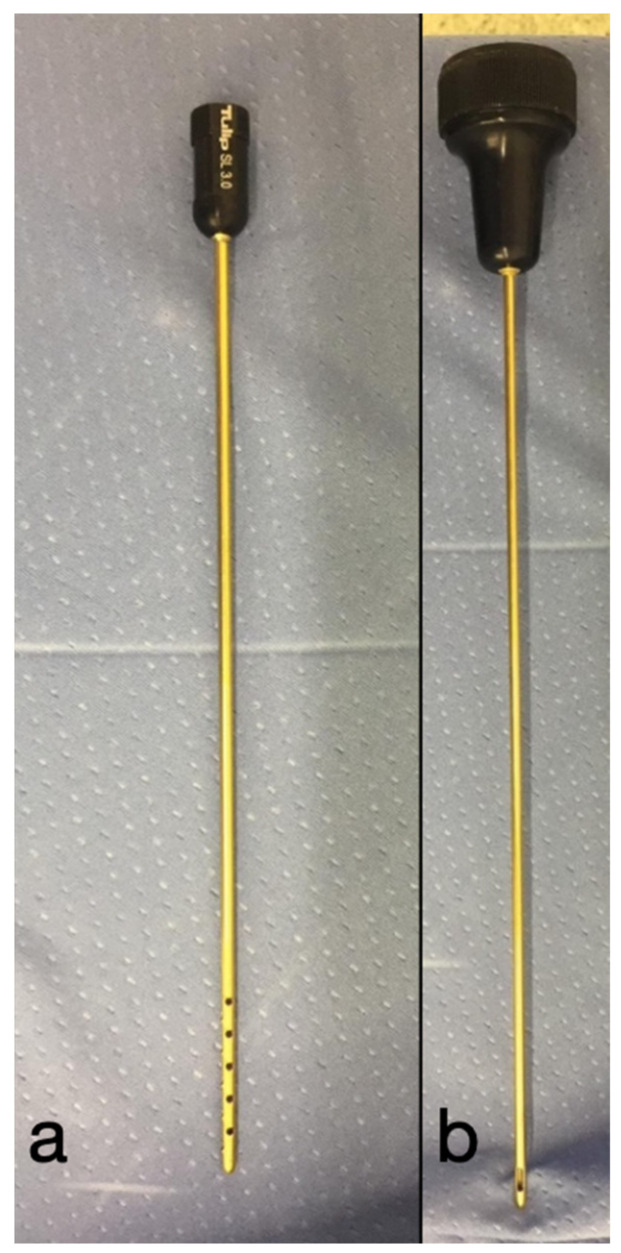
(**a**) The 2.8 mm blunt infiltration cannula and (**b**) 3.8 mm blunt liposuction cannula.

**Figure 2:**
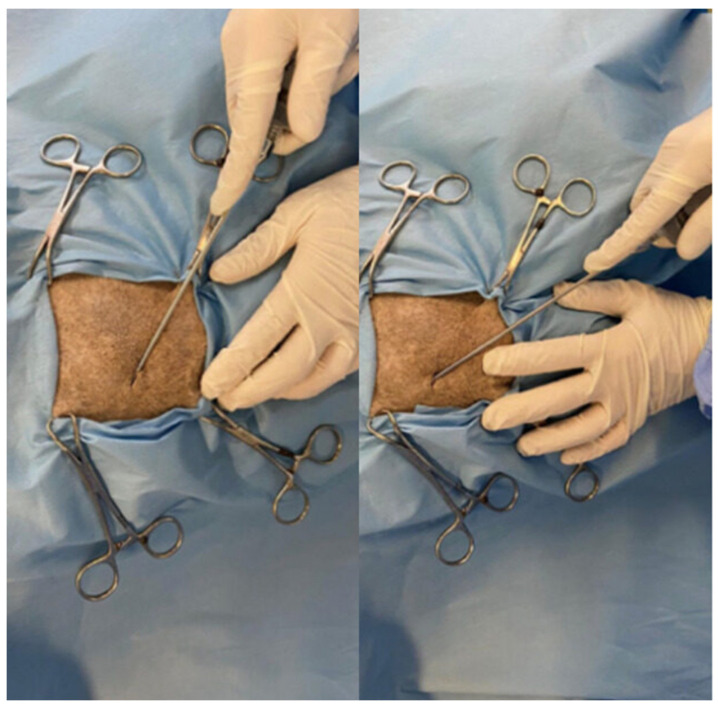
Adipose tissue sampling from the lumbar region.

**Figure 3:**
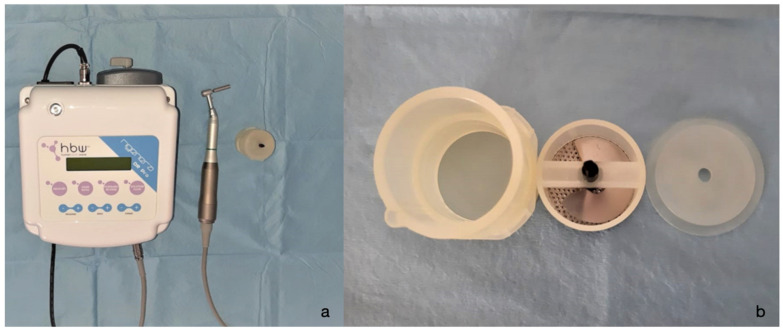
(**a**) Motorized apparatus of the Rigenera^®^ system; (**b**) RigeneraCons^®^ sterile capsule.

**Figure 4:**
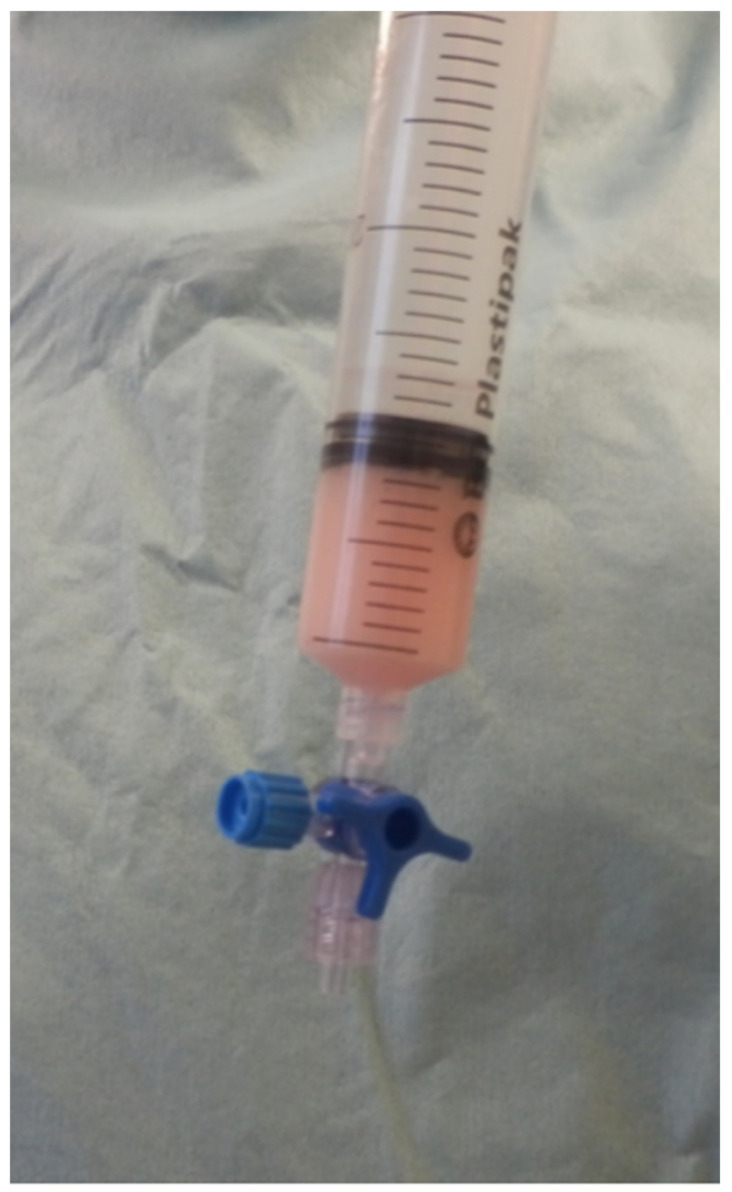
Microfragmented adipose tissue graft (MFAT) obtained from the dog, ready to use.

At T0, the patients of this study group received the Rigenera^®^ (HBW, Turin, Italy) treatment. After anesthesia, trichotomy was performed in the region of the lumbar spine, taking the fifth lumbar vertebra as anatomical reference. Each dog was placed in sternal recumbency, and the surgical site’s skin was antiseptically prepared. After a normal sterile dressing, the surgeon made an incision with a #15 scalpel blade, which allowed the use of a blunt infiltration cannula (Figure 1). The preparatory infiltration of the adipose tissue was performed using a surgical fanning technique on different planes, taking care to homogeneously distribute the tumescent Klein’s solution into the underlying fat. After a few minutes, a blunt liposuction cannula was used, equipped with a 50 mL Luer-Lock syringe under suction. The same surgical fanning technique was used during lipoaspiration (Figure 2). Once the adipose tissue was obtained, it was homogenized and mixed between two syringes connected via a three-way stopcock. The subsequent procedure required a resting phase for the tissue in order to separate the liquid part from the adipose part, which was brought into suspension. The liquid part was subsequently discharged, and the homogenized adipose tissue was inserted into the proper RigeneraCons^®^ (HBW, Turin, Italy) (Figure 3). The RigeneraCons^®^ device consists of a plastic container (a sterile capsule), inside which the tissue is broken up into micrografts of about 80 microns by rotating a helix positioned on a grid consisting of microblades and calibrated holes. After disaggregation, a 10 mL syringe was connected to the proper syringe fitting port of the RigeneraCons^®^, obtaining a microfragmented adipose tissue graft (MFAT). MFAT was subsequently used intra-articularly in the patient’s pathological joint; approximately 1–2 mL of MFAT was used for each joint (Figure 4).

### 2.7. Canine Brief Pain Inventory

The Canine Brief Pain Inventory (CBPI) [40] allows owners to rate the severity of the pain they perceive their dog to be experiencing and the degree of influence this pain has on the dog’s quality of life. This questionnaire contains four items relating to the severity of the dog pain and six items describing how that pain interferes with the dog’s daily activities, for a total of 10 items for the owner to answer. Each CBPI pain item is presented with numerical rating scales from 0 to 10, where 0 represents no pain, and 10 represents extreme pain. In the same way, for the interference items, 0 represents no interference, and “10” represents complete interference. In this clinical study, the CBPI was used as an outcome, submitted and completed by the owner during the trial at T0, T1, T2, and T3.

### 2.8. Clinical Examination

The head orthopedic surgeon of the University of Camerino performed this part of the examination, in which the completion of a specific clinical form based on the Vesseur modified lameness classification system [41] was requested. This clinical card contained several fields to be filled in using a numerical scale from 1 (no clinical evidence of lameness) to 5 (non-weight-bearing lameness). The fields were divided into lameness during the walking phase, in stance, pain on palpation, and evaluation of the contralateral limb. For each dog, the specialist compiled the patient’s pain sensation during a preliminary inspection using the visual analogue pain (VAS) scale [42], which has the same numerical severity scale as the CBPI, from 0 to 10. The clinical card examination also included a measurement of the range of motion of the affected joint, measured by orthopedic goniometer; the limb circumference, measured by a tape measure; and the Body Condition Score (BCS) of each patient. This clinical evaluation was used as the clinical outcome and was performed by the head orthopedic surgeon at time T0 (before treatment) and at T1, T2, and T3 after treatment for each group.

### 2.9. Radiographic Examination

The radiographic examination served to confirm the diagnosis of OA and to obtain orthogonal projections of the joint involved in the pathological process. The study was reported by the head of the Radiology Unit of the Veterinary Teaching Hospital of the University of Camerino, who attributed blindly for each patient a degree of OA according to a modified Kellgren–Lawrence scale [39], considering different radiographic signs, including the presence of osteophytes, bone sclerosis, joint narrowing and/or incongruence, and the presence of capsular ectasia (Table 2)**.** The degree of OA was attributed by means of a numerical scale from 0 (absence of OA) to 4 (highest degree of OA), established on the basis of the interval determined by the calculation of the total score. This radiographic scoring system designated a degree of OA for each patient at T0, allowing researchers to evaluate its evolution following the treatment after 30 and 60 days (T1, T2).

### 2.10. Synovial Fluid Examination

At the time of treatment (T0) and after 30 (T1) and 60 (T2) days, an aliquot of synovial fluid was taken for cytological examination. In the Veterinary Pathology Unit of the University of Camerino, after a general evaluation of the concentrations and aspect of the fluid, a cytological assessment was blindly performed in an attempt to obtain a degree of OA, considering the cytological concentration, the inflammatory cellular types, and the presence and modifications of cells belonging to synovia, cartilage, and eventually bone. In order to generate a cytological score, various parameters were considered, including the prevalence of inflammatory cells, the presence and the morphology of synovial cells, cartilage fragments, blood contamination, and sliding pattern related to the quality of the matrix. For each parameter evaluated, a numerical score from 1 to 3 was assigned on the basis of the progressive severity of the parameter. As reported in Table 3, a combination of these different gradings produced a final score for each sample ranging from 1 to 4. Briefly, each value reflected the description of the synovial fluid evaluated according to the following interpretation: 1 = paraphysiological; 2 = mild; 3 = moderate; and 4 = severe inflammation of the synovium. This system allowed the pathologist to establish for each patient of each group an initial degree of joint inflammation at T0 and to evaluate its evolution during the clinical trial at T1 and T2 (Table 3).

### 2.11. Synovial Cytokine Assay

After cytological assessment, the remaining synovial fluid aliquots, obtained from dogs sampled at T0, T1, and T2, were used to evaluate the concentration of three cytokines (TNFα, IL-6, and IL-1β) through Nori^®^ canine ELISA kits (Genorise Scientific Inc., Glen Mills, PA, USA), according to the manufacturer’s protocols. These three biomarkers were chosen based on previous literature supporting the constant presence of these three cytokines and their concentration as relating to OA severity [43,44,45,46,47]. In our study, each aliquot of synovial fluid was used first for TNF-α evaluation and subsequently for IL-6 and IL-1β.

### 2.12. Statistical Analysis

Data from all the evaluations during the study were pooled and reported with their arithmetic means and standard deviations. Ordinal variables were analyzed and compared between the two groups with the Mann–Whitney test. Friedman’s test, followed by Dunn’s post hoc test, was also used to compare the timepoints within each group. Considering the anatomical differences between different limbs and joints, the variables of limb circumference and ROM were normalized by taking the value of T0 as a reference point and transforming the absolute variations calculated at T1 and T2 into relative variations (percentage variation between times, %Var). The %Var values were then analyzed and compared between groups with a Student’s t-test and compared between times within each group with a repeated measures analysis of variance followed by the Holm–Sidak post hoc test. Fisher’s exact tests were used for frequency analysis (dogs requiring administration of anti-inflammatory drugs). Differences with *p*-values < 0.05 were considered statistically significant. All data were analyzed with Prism 8 for MacOS software, version 8.2.1 (GraphPad Software Inc., San Diego, CA, USA).

## 3. Results

### 3.1. Enrolled Patients

Forty pet dogs belonging to different breeds and affected by OA met the eligibility criteria and were randomly divided into two groups: the MFAT group that received the Rigenera^®^ treatment, and the hyaluronic group that received HA (Table 4). Dogs selected for each group were aged between 3 and 13 years, with a mean of 7.5 for the MFAT group and 8 for the hyaluronic group; the mean weight was 29.1 kg, associated with a body condition score (BCS) of 5.2, for the MFAT group, and 26.1 kg, associated with a BCS of 5.8 for the HA. No restrictions were placed on sex nor the affected joint. Gender was distributed randomly as follows: 6 males (1 neutered) and 14 females (9 neutered) for the MFAT group, and 10 males (2 neutered) and 10 females (5 neutered) for the Hyaluronic group.

The affected joints were, respectively, seven shoulders, six elbows, four hips, and three stifles for the MFAT group and six shoulders, four elbows, three hips, six stifles, and one tarsus, for the hyaluronic group. No statistically significant difference between groups was present for the inclusion criteria of weight, BCS, age, and sex, and there were no statistically significant differences between the groups concerning clinical examination, CBPI, and radiographic evaluation. Arthrocentesis produced an adequate volume of synovial fluid for cytological evaluation in 35 of 40 dogs at T0. However, the volume was very challenging for the cytokines assay, being adequate for only 11, 6, and 3 of the 40 patients for TNFα, IL-6, and IL-1β, respectively. No statistically significant differences were detected for the cytological score at T0 between the groups, and the sample size for the cytokine assay was not adequate for statistical analysis.

### 3.2. Short-Term Outcome

#### 3.2.1. Clinical Examination

**Figure 5:**
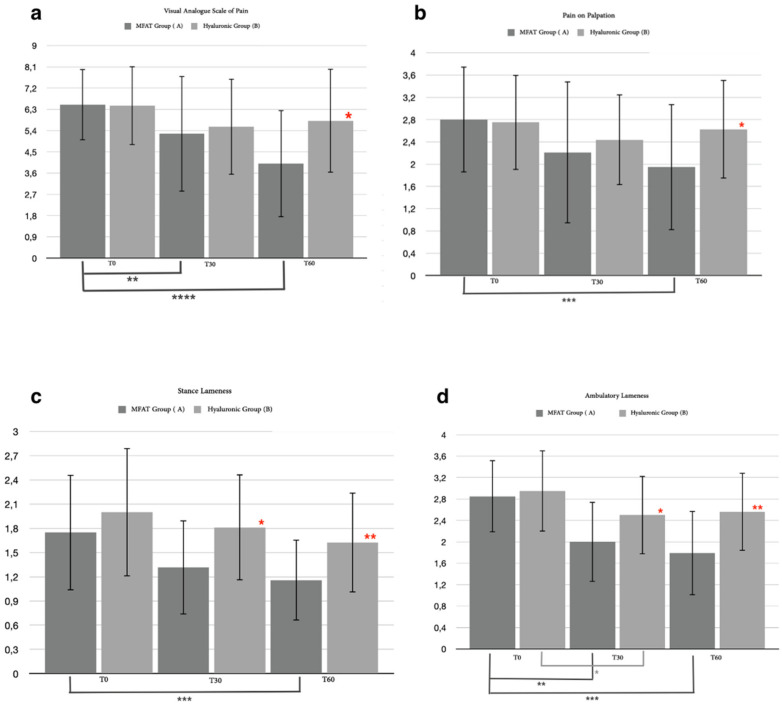
These graphs show the differences in statistical analysis data between hyaluronic group and MFAT group for (**a**) visual analogue score of pain after 30 and after 60 days. Red asterisks (*: *p* = 0.0149) indicate significant differences between groups, and group-colored asterisks indicate significant differences within each group (**: *p =* 0.0094; ***: *p* = 0.0001); (**b**) pain on palpation after 30 and after 60 days. Red asterisks (*: *p* = 0.0372) indicate strong significant differences between groups, and group-colored asterisks indicate significant differences within each group (***: *p* = 0.0002); (**c**) stance lameness after 30 and after 60 days. Red asterisks (*: *p* = 0.0247; **: *p* = 0.0087) indicate significant differences between groups, and group-colored asterisks indicate significant differences within each group (***: *p* = 0.0006); and (**d**) ambulatory lameness after 30 days and after 60 days. Red asterisks (*: *p* = 0.0338; **: *p* = 0.0037) indicate significant differences between groups, and group-colored asterisks indicate significant differences within each group (*: *p* = 0.0077; **: *p* = 0.0027; ***: *p* = 0.0001).

We identified 35 out of 40 patients (87.5%) who completed the short-term evaluation period after 60 days at T2: 19 out of 20 (95%) for the MFAT group and 16 out of 20 (80%) for the hyaluronic group (Table 5). The excluded patients (one for the MFAT group and four for the hyaluronic group) had already required the use of systemic NSAIDs after thirty days due to an aggravation of pain. The statistical analysis of the 35 patients for the walking lameness score showed significant differences between the A and the B groups after 30 days (*p* = 0.0338) and after 60 days (*p* = 0.0037). Friedman’s test within the groups showed a statistical difference for the hyaluronic group only between T0 and T1 (*p* = 0.0077), while for the MFAT group, the Friedman test revealed an important statistically significant difference between T0 and T1 (*p* = 0.0027) and between T0 and T2 (*p* = 0.0001) (Figure 5d). For the stance lameness score, the statistical analysis showed significant differences between the MFAT group and the hyaluronic group after 30 days (*p* = 0.0247) and after 60 days (*p* = 0.0087). A statistical analysis within the groups showed a significant difference only for the MFAT group (*p* = 0.0006) between T0 and T2 (Figure 5c). A Mann–Whitney statistical test for the pain on palpation score showed mild significant differences between the MFAT and hyaluronic groups after 60 days (*p* = 0.0372) and also showed a strong statistical difference (*p* = 0.0002) within the MFAT group between T0 and T2 (Figure 5b). A statistical analysis of the visual analogue score of pain proved there was a significant difference between the groups at T2 (*p* = 0.0149) associated with a strong significance within the MFAT group between T0 and T2 (*p* < 0.0001) and T0 and T1 (*p* = 0.0094) (Figure 5a). No statistically significant differences were noted between groups for BCS, with only one difference within the hyaluronic group (*p* = 0.0047) between T0 and T2. For the statistical analysis of circumference and ROM, no statistical differences in relative variation were reported either between or within groups.

#### 3.2.2. Canine Brief Pain Inventory Index (CBPI)

**Figure 6:**
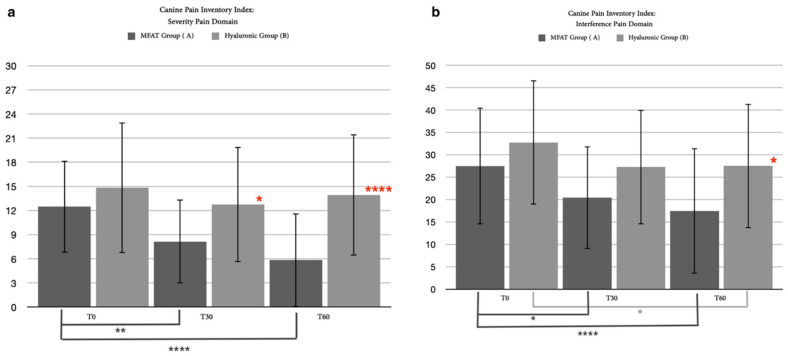
These graphs show the differences in statistical analysis data between Hyaluronic group and MFAT group for (**a**) CBPI pain severity after 30 days and after 60 days. Red asterisks (*: *p* = 0.0154; ****: *p* = 0.0133) indicate significant differences between groups, and group-colored asterisks indicate significant differences within each group (**: *p* = 0.0094; ****: *p* < 0.0001); and (**b**) CBPI pain interference after 30 days and after 60 days. Red asterisks (*: *p* < 0.0001) indicate significant differences between groups, and group-colored asterisks indicate significant differences within each group (*: *p* = 0.0021; ****: *p* < 0.0001).

In addition to the clinical examination, 35 out of 40 patients completed the short-term follow-up period for CBPI: 16 for the hyaluronic group and 19 for the MFAT group. The resulting data derived from the questionnaire were divided into two domains, the pain severity domain (PSI) and the pain interference domain (PII), as is usually used for the CBPI. The statistical analysis for the PSI proved there was a mild significant difference between the MFAT group and the hyaluronic group after 30 days (*p* = 0.0236) and a strong significant difference after 60 days (*p* < 0.0001). Friedman’s test did not show a significance for any time for the hyaluronic group, while within the MFAT group, there was an important significant difference between T0 and T1 (*p* = 0.0021) and between T0 and T2 (*p* < 0.0001) (Figure 6b). For the PII, a Mann–Whitney test between the MFAT group and the hyaluronic group showed a significant difference (*p* = 0.0154) after 60 days; a significant difference was detected within T0 and T2 for the hyaluronic group (*p* = 0.0133), and between T0 and T1 (*p* = 0.0094) and between T0 and T2 (*p* < 0.0001) within the MFAT group (Figure 6a).

#### 3.2.3. Radiographic Assessment

**Figure 7:**
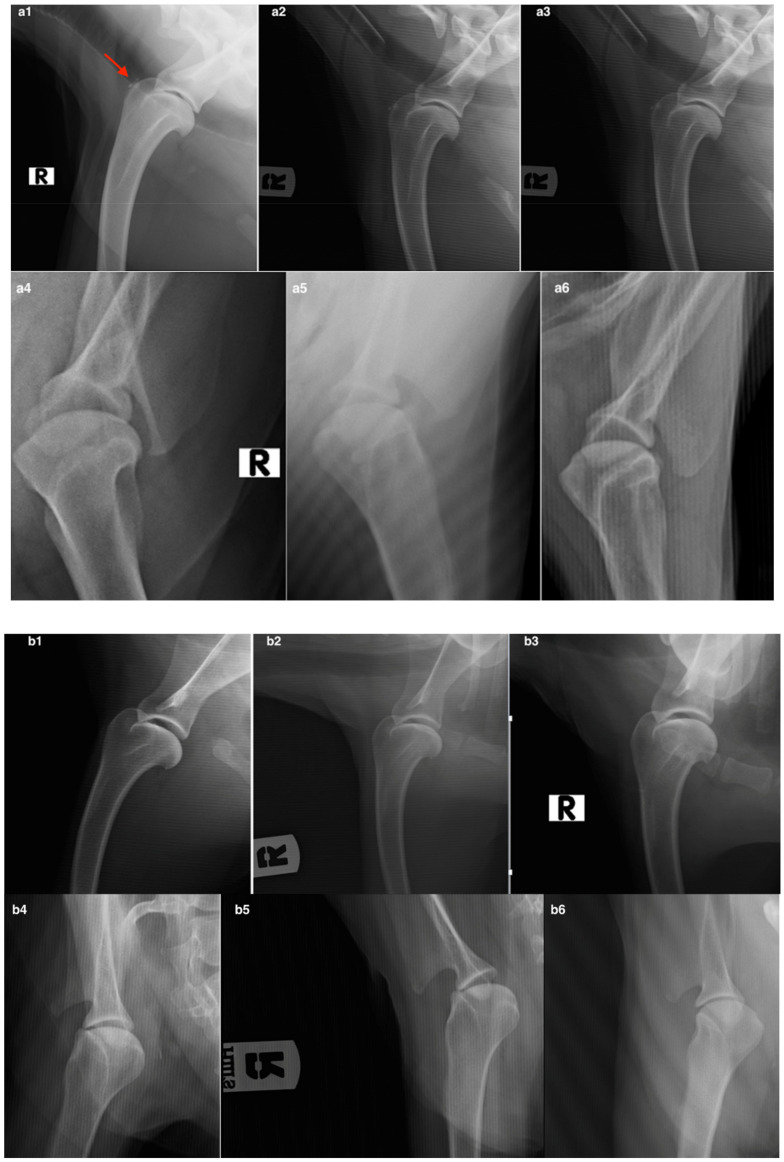
The figure shows the radiographic examination performed in the MFAT group at T0 (**a1**–**a4**), T1 (**a2**–**a5**), and T2 (**a3**–**a6**) and in the Hyaluronic group at T0 (**b1**–**b4**), T1 (**b2**–**b5**), and T2 (**b3**–**b6**). Figure (**a1**–**a6**): X-ray images of a patient of the MFAT group show a picture of chronic shoulder arthrosis with the presence of osteophytes (red arrow) (**a1**). Figure (**b1**–**b4**) shows a radiographic picture of chronic shoulder osteoarthritis of a patient in the control group at T0. Figures (**b2**–**b5**) and (**b3**–**b6**) show the evolution of the radiographic picture at the assessment times T1 and T2 compared to T0.

**Figure 8:**
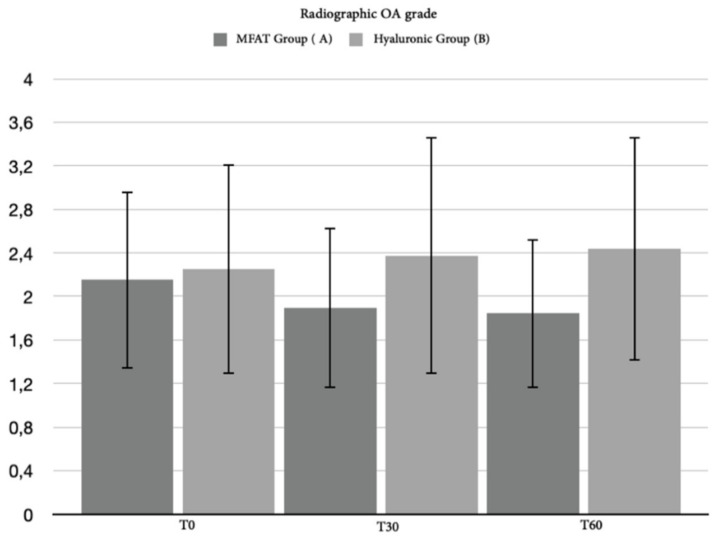
These graphs show the differences in statistical analysis data between hyaluronic group and MFAT group for the radiographic OA grade after 30 days and after 60 days. As shown by the absence of asterisks, no statistical difference between groups, either within groups was highlighted by the statistical analysis.

As with the clinical examination and the CBPI index, we collected radiographic data for 35 out of the 40 patients. Although a slight worsening in the radiographic images of all patients was highlighted, due in particular to a slight increase in the size of the osteophytes, the statistical analysis did not reveal any significant differences between the groups or within the group (Figure 7 and Figure 8).

#### 3.2.4. Synovial Assessment

**Figure 9:**
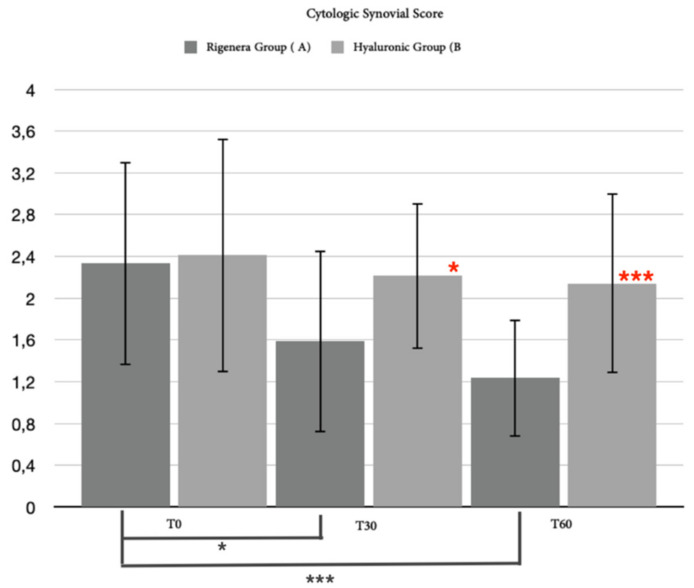
This graph shows the differences in statistical analysis data between hyaluronic group and MFAT group for the cytological synovial score after 30 days and after 60 days. Red asterisks (*: *p* = 0.0331; ***: *p* = 0.008) indicate significant differences between groups, and group-colored asterisks indicate significant differences within each group (*: *p =* 0.0258; ***: *p* = 0.0006).

**Figure 10:**
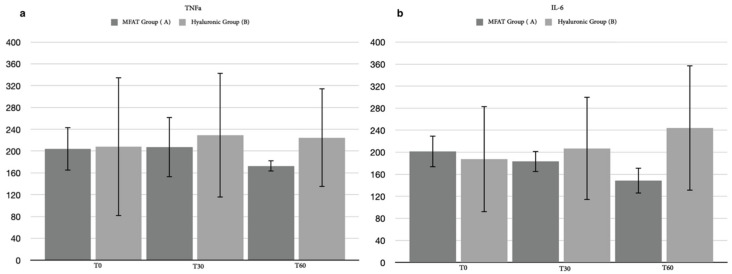
Difference of the necrosis tumor factor α (TNFα) (**a**) and interleukin-6 (IL-6) (**b**) between hyaluronic group and MFAT group after 30 days and after 60 days.

In 35 of the 40 patients enrolled in the study (87.5%), it was possible to obtain an adequate amount of synovial fluid before treatment (T0) to cytologically evaluate and classify the inflammatory state of the joint. Of these, only 31 patients (88.57%) completed the short-term follow-up (T2): 14 for hyaluronic group and 17 for the MFAT group. Statistically, in 31 patients, the cytological synovial score showed a mild significant difference between groups after 30 days (*p* = *0*.0331) and a strong significant difference after 60 days (*p* = 0.0008). No differences were observed within the hyaluronic group, but a significant difference was observed between T0 and T1 (*p* = 0.0258) and T0 and T2 (*p* = 0.0006) in the MFAT group (Figure 9). In general, the lack of adequate volume of synovial fluid made it difficult to perform the synovial cytokine assay. The raw data obtained by the ELISA tests were not sufficient to perform statistical analyses for TNFα, IL-6, or IL-β. We collected useful data during the short follow-up for 7 out of 40 patients for TNFα (17.5%), 4 for the MFAT group and 3 for the hyaluronic group; 5 out of 40 for IL-6 (12.5%), 2 for the MFAT group and 3 for the hyaluronic group; and only one patient for IL-1β (2.5%), belonging to the hyaluronic group. Despite no statistical analysis, the ELISA tests suggested a mild decrease in terms of cytokine concentration in the MFAT group, while it showed an opposite trend in the hyaluronic group (Figure 10).

### 3.3. Long-Term Outcome

#### 3.3.1. Clinical Examination

**Figure 11:**
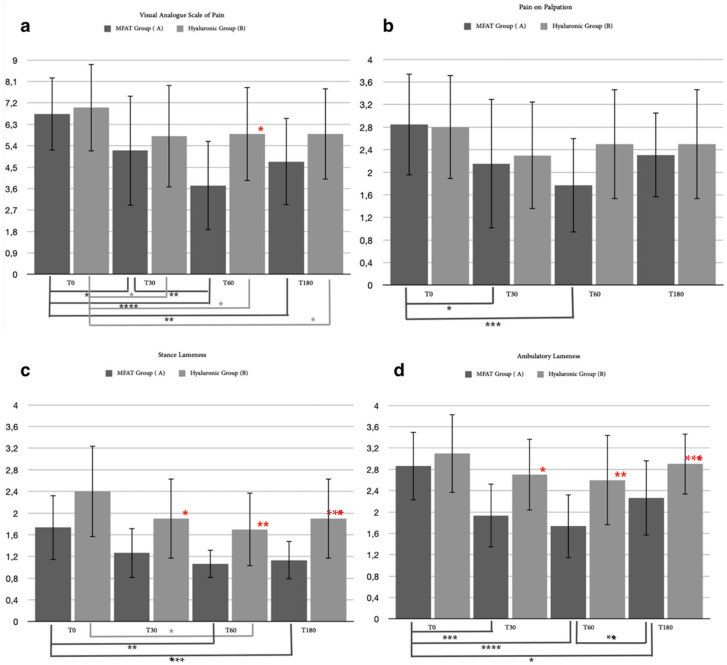
Differences in statistical analysis data between hyaluronic group and MFAT group for (**a**) visual analogue score of pain after 30 days, 60 days, and 180 days. Red asterisks (*: *p* = 0.01) indicate significant differences between groups, and group-colored asterisks indicate significant differences within each group (*: *p* < 0.05; **: *p* < 0.01; ****: *p* < 0.0001); (**b**) pain on palpation after 30 days, 60 days, and 180 days. Group-colored asterisks indicate significant differences within each group (*: *p* = 0.0109; ***: *p* = 0.0002); (**c**) stance lameness after 30 days, 60 days, and 180 days. Red asterisks (*: *p* = 0.0259; **: *p* = 0.0059; ***: *p* = 0.0064) indicate significant differences between groups, and group-colored asterisks indicate significant differences within each group (*: *p* = 0.0464; **: *p* = 0.0089; ***: *p* = 0.0196); and (**d**) ambulatory lameness after 30 days, 60 days, and 180 days. Red asterisks (*: *p* = 0.0118; **: *p* = 0.0094; ***: *p* = 0.0413) indicate significant differences between groups, and group-colored asterisks indicate significant differences within each group (*: *p* = 0.0339; **: *p* = 0.0477; ***: *p* = 0.0007; ****: *p* < 0.0001).

Only 25 patients out of the 35 (71.43%) who completed the short-term follow-up concluded the study with a long-term follow-up after 180 days of treatment (T3): 15 of 19 (78, 94% of the MFAT group patients) for the MFAT group and 10 out of 16 (62.5% of the hyaluronic group patients) for the hyaluronic group. The patients that were not included (four for the MFAT group and six for the hyaluronic group) required the use of systemic NSAIDs between 60 and 180 days due to an aggravation of joint pain. A statistical analysis was performed with clinical examination data relating to the short-term follow-up associated with the 6-month follow-up data of the 25 patients, both between and within groups. Mann–Whitney’s test for ambulatory lameness showed a statistically significant difference between groups at 30 days (*p* = 0.0118) and after 60 days (*p* = 0.0094), which persisted at 180 days (*p* = 0.0413); no statistical significances were noticed within the hyaluronic group, while there were significant differences in the MFAT group between T0 and 30 days (*p* = 0.0007), T0 and 60 days (*p* < 0.0001), T0 and 180 days (*p* = 0.0339), and between 60 and 180 days (*p* = 0.0477) (Figure 11d). An investigation of stance lameness showed a significant difference between groups at T1 (*p* = 0.0259), T2 (*p* = 0.0059), and T3 (*p* = 0.0064); a statistical difference was noticed within the hyaluronic group between T0 and T2 (*p* = 0.0464) as well as in the MFAT group between T0 and T2 (*p* = 0.0089) and between T0 and T3 (*p* = 0.0196) (Figure 11c). Mann–Whitney’s test showed no significance between the groups considering pain on palpation, which was noticed only within the MFAT group between T0 and 30 days (*p* = 0.0109) and between T0 and 60 days (*p* = 0.0002) (Figure 11b). A statistical analysis between the MFAT and hyaluronic groups proved a significant difference for the VAS of pain only after 60 days from treatment (*p* = 0.0100); statistical testing showed a significance within the groups between day 0 and any other follow-up in both groups (hyaluronic: T0–T1, *p* = 0.0464; T0–T2, *p* = 0.0304; and T0–T3, *p* = 0.0377; MFAT: T0–T1, *p* = 0.0404; T0–T2, *p* < 0.0001; T0–T3, *p* = 0.0015) and within the MFAT group between 30 and 60 days (*p* = 0.0037) (Figure 11a). No statistical evidence was found between the groups for the BCS of 25 patients at any time, while a significant difference was noted with respect to T0 and T2 within both groups (hyaluronic: *p* = 0.0050; MFAT: *p* = 0.0381).

#### 3.3.2. Canine Brief Pain Inventory Index

**Figure 12:**
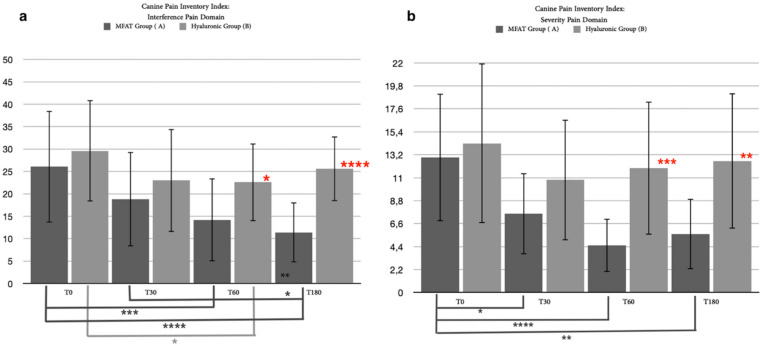
Differences in statistical analysis of data between the hyaluronic group and the MFAT group for (**a**) the CBPI for pain interference after 30 days, 60 days, and 180 days. Red asterisks (*: *p* = 0.0317; ****: *p* < 0.0001) indicate significant differences between groups, and group-colored asterisks indicate significant differences within each group (*: *p* = 0.0335; **: *p* = 0.0179; ***: *p* = 0.0002; ****: *p* < 0.0001; and (**b**) the CBPI for pain severity after 30 days, 60 days, and 180 days. Red asterisks (**: *p* = 0.0025; ***: *p* = 0.0004) indicate significant differences between groups, and group-colored asterisks indicate significant differences within each group (*: *p* = 0.0281; **: *p* = 0.0011; ****: *p* < 0.0001).

Another long-term result was imported from the questionnaire completed by the owners. Similar to the clinical evaluation, we collected the results of 25 of the 35 patients who completed the short study period. The statistical analysis resulting from the PSI between groups showed significant differences after 60 (*p* = 0.0004) and 180 days (*p* = 0.0025), associated with a significant difference within the MFAT group between day 0 and any other day (T1, *p* = 0.0281; T2, *p* < 0.0001; T3, *p* = 0.0011) (Figure 12b).

The PII score resulted in statistically significant differences between the groups at T2 (*p* = 0.0317) and T3 (*p* < 0.0001), also showing a significancy within the hyaluronic group between days 0 and 60 (*p* = 0.0335) and within the MFAT group between T0 and T2 (*p* = 0.0002), T0 and T3 (*p* < 0.0001), and between T1 and T3 (*p* = 0.0179) (Figure 12a).

#### 3.3.3. Excluded Patients

Some patients were excluded during the research period, in particular, 5/40 (12.5%) for the short-term outcome (10% of the hyaluronic group and 2.5% of the MFAT group), which rose to 15/40 (37.5%) for the long-term outcome: 25% for the group hyaluronic and 12.5% for the MFAT group. Considering the percentage of patients who abandoned the present research due to inadequate pain control through intra-articular therapy, the authors wanted to evaluate the presence of statistical differences in the frequency of the administration of anti-inflammatories at different follow-up terms. Fisher’s exact test found no statistically significant differences between the groups.

## 4. Discussion

Osteoarthritis is currently considered to be one of the world’s most significant challenges for both animal and human health systems [46,47,48]. This places OA among the most investigated conditions for the mutual co-study of animals and humans. The foundation of this concept was introduced in One Health Medicine, where human health was recognized as having a close connection with veterinary health. In particular, the link with the animal model becomes very close with the canine species. Considering the high incidence of OA in the canine species in relation to the size of the population and taking into account the dog’s lifespan, which is equivalent in stages to humans, and the fact that both species share the same comorbidities, such as obesity, and undergo identical treatments, such as anti-inflammatory drugs or joint replacement surgery, the dog is probably the closest to a gold-standard animal model for OA currently available. In addition, the progression of OA in the canine species develops in a similar way to its pathology in humans. This interspecies connection allows a longitudinal evaluation of both the disease and its therapy in cases of spontaneous OA [49,50,51].

Extensive research has been conducted in recent decades to treat OA, or at least to slow its progression. The exponential increase in the literature on regenerative medicine, with a particular focus on advances regarding stem cells, and the evaluation of the characteristics and uses of crude isolated tissue, such as bone marrow concentrate or stromal vascular fraction (SVF) from adipose tissue, has recently been shown to be an acceptable therapeutic treatment for many diseases as well as for orthopedic conditions. Many human clinical case reports have demonstrated the efficacy of the intra-articular administration of microfragmented adipose tissue (MFAT) for spontaneous OA in different joints [51,52,53,54,55,56]. The establishment of the concept of niche matrix and minimal mechanical manipulation of the tissue to support the gene expression pattern and the efficacy of mitigating the inflammatory cascade is likely responsible for the clinical success of treatments with MFAT. In addition, this notion offers new frontiers for biomedical engineering, which increasingly introduces new devices that provide automated tissue disaggregation systems [35,57,58,59,60]. The Rigenera^®^ micrografting technology has recently been seen as a promising system for rapidly harvesting various disaggregated tissues, as a source of MFAT, to be used for many different pathologic processes [14,35,61,62,63,64,65]. The principal use of this technology was investigated in plastic surgery, with an increasing trend in a focus on the orthopedic field [66,67,68,69,70,71,72,73,74,75,76]. In the veterinary field, Palumbo Piccionello et al. [64] demonstrated the effectiveness of the use of MFAT, obtained through the Rigenera^®^ technology, in the repair of tendon lesions on an ovine model. The results of the aforementioned study suggested that MFAT, in addition to exerting an anti-inflammatory effect, stimulated and favored the deposition of tendon fibers organized according to a texture more similar to the physiological one of the tissue, compared to untreated tendons. Furthermore, the elastosonographic evaluation showed that the tendons treated with MFAT had a lower degree of stiffness than the untreated ones [64]. The in vitro validation of the Rigenera^®^ system for MFAT in the canine species was obtained previously, allowing the consequent prospective clinical evaluation in vivo [77].

The results obtained from our study showed an interesting statistically significant difference in evaluated parameters, both for the short (30–60 days) and long-term outcome (180 days), connected with a symptomatic improvement, which persevered longer for the MFAT-treated group (MFAT group). The two study groups were compared by evaluating the trend of six clinical parameters, specifically the degree of lameness, the perceived pain, the circumference of the affected limb, the degree of range of motion (ROM), the degree of arthrosis highlighted radiographically, the BCS, and cytological assessment of the synovial fluid. These six clinical items evaluated during each time of the research (T0–T3) showed superior efficacy for the treatment with adipose micrografts within the patient and when compared between the groups over 180 days. The graphical representation of the short-term clinical outcome showed a good decrease in the parameters evaluated after 30 days in both therapies, while it showed differences between treatments at the evaluation after 60 days. The clinical improvement continued and persisted better for the MFAT group. Similar trends were observed between groups for long-term follow-up after 180 days although connected to gradual increases of lameness and pain in both.

The degree of lameness in the short term improved for both groups at 30 days, while in the evaluation at 60 days, there were considerable differences between groups. The MFAT group showed further clinical improvement in lameness score after 60 days of treatment, while in the control group, the degree of lameness remained unchanged. The significant difference between the study groups persisted for the lameness score in the long-term clinical outcome. Clinical efficacy loss over time was supposed in the control group. This scenario is widely supported by veterinary literature due to the temporary anti-inflammatory action of viscosupplementation, especially after a single infiltration of hyaluronic acid. Repeated intra-articular infiltrations of hyaluronic acid have therefore been recommended by most authors [78,79,80]. However, a single administration of HA in our study was necessary to compare the difference between these intra-articular treatments properly. The greater efficacy in terms of reduction of the degree of lameness of the MFAT treatment in the long term, compared to the treatment with single infiltration of hyaluronic acid, was also confirmed by the results obtained by Zeira et al. [34] in a study aimed at evaluating the effectiveness of the use of micrografts of autologous adipose tissue, subjected to mechanical disintegration, in the treatment of OA in dogs. Of the 130 patients undergoing treatment, 88% showed a gradual improvement in clinical symptoms up to 6 months after treatment, and only 1% showed a worsening of symptoms [34].

In our study, owners reported an improvement for their dogs with regard to the severity of pain and its interference with the quality of life in both groups although data showed a greater significance in subjects treated with purified microfragmented adipose tissue. Crucial differences were noticed between groups for the short-term outcome, detected through pain severity at T2 (*p* < 0.0001) with solid evidence of a reduction of pain interference within the MFAT group (T0–T2, *p* < 0.0001). Similarly, the long-term outcome showed a substantial decrease in pain severity and interference for the MFAT group supported by a statistical analysis and a statistical significance for pain interference after 180 days (*p* < 0.0001). As in the literature on human medicine, an improvement in pain perception is validated in both the short and long term following joint infiltration with MFAT [81,82,83,84]. Additionally, recent veterinary literature suggests evidence of clinical pain control over 180 days due to intra-articular administration of autologous purified and microfragmented adipose tissue to treat OA in dogs [34,81]. One of the authors’ main concerns was the impracticality of making the owner and clinician blind for the treatment group to which the patients belonged [85,86]. The same issue was encountered for the clinician’s assessment due to the lack of an analytical evaluator of lameness, such as a force plate system for gait analysis. At the same time, other criticalities are related to the patient’s pain assessment. Regardless, the clinical efficacy of MFAT in pain control that we observed in our study has been highlighted in several other studies, both in humans and veterinary medicine [34,51,52,53,54,55,56,81]. Recently, a systematic review of the literature on human medicine has shown how the administration of intra-articular adipose stem cells, despite the different studies proposed over time, has an exclusive role in the control of joint pain without showing objective improvements in the cartilage surface or OA progression [87]. The similarities that were observed in the literature on the use of cultured-expanded stem cells and cellular microfragments greatly emphasize the curiosity about the mechanism of action of these therapies, increasing the focus on the paracrine and immunomodulatory effect of the stem niche rather than for the regenerative role of mesenchymal stem cells.

As expected by the authors, there were no statistical differences in circumference, ROM, or BCS over two months for both groups. In particular, considering the assumed importance of body weight in the management of OA [88,89,90], the variation in the BCS of both groups was evaluated in order to determine whether the clinical improvements could be affected by significant changes in body weight between study times. The slight, not statistically significant variation highlighted after six months for BCS in both groups between 0 and 60 days (*p* < 0.05) was likely related to the patients’ inhomogeneity, which altered the data. This statistical nonrelevance of the BCS parameter allows us to affirm that the results obtained in the study are attributable only to the therapeutic efficacy of the treatments administered.

The most interesting data were obtained from the cytological analysis of the synovial fluid, indicating an improvement of inflammation grade for both groups and showing statistically significant differences in support of the MFAT group with respect to the hyaluronic group at day 30 and strengthening after 60 days. Furthermore, the statistical analysis showed an exclusive significance within the group treated with adipose MFAT, which was enhanced after 60 days. In particular, 76.5% of patients treated with MFAT injection improved their cytological score at T2. To the best of our knowledge, this is the first study appraising synovial fluid after the administration of microfragmented adipose tissue in dogs. The cytological analysis of synovial fluid provides a qualitative measure of joint health, mainly associated with prognostic purposes [91,92,93,94]. In addition to the short-term cytological evaluation, the concentration of inflammatory cytokines in the synovial fluid was also evaluated. Although it was not possible to perform a complete statistical analysis due to the lack of samples, the trend of data indicated a reduction in cytokine concentration in patients treated with MFAT when compared with the hyaluronic group, in which an opposite trend was observed. A positive correlation between cytokines concentration in synovial fluid and OA degree in dogs was described by Allen et al., even without clinical correlation of force plate outcome with lameness [92]. Thus, the predictive value of these biomarkers is not yet clearly understood.

Radiographic assessment showed no differences between the groups nor within the groups over the follow-up. This evaluation was in agreement with the veterinary literature on intra-articular treatment using MFAT [81].

The entire cohort of patients, who concluded the study, had to follow a management therapy, limiting uncontrolled activities, favoring leashed walks, and avoiding nutraceuticals or NSAIDs. Patients that required anti-inflammatories administration were excluded from the research, causing inconsistencies between groups in both short and long-term outcomes. These inconsistencies have been accentuated over time. The resulting number of excluded patients was very different between the treatment groups; in fact, 50% of patients in the control group needed to use NSAIDs within six months, compared to 12.5% in the MFAT group. Although there is no statistical evidence, the data obtained proved to be very interesting in supporting treatment with adipose micrografts. No statistically significant differences were found between the groups at T0, despite the randomization of patients, considering different sexes, ages, weights, and joints affected by OA.

Our study highlighted some limitations related to the eligibility criteria: various dog sizes and joints influenced the clinical parameters assessed by the veterinarian and the pet owner. For example, with the same OA grade, we observed higher pain severity scores in patients who demonstrated reduced weight-bearing at the station, such as those with stifle versus elbow arthropathy. These differences highlighted in our study are in agreement with Pavarotti and colleagues. Despite observing the control of pain and lameness in patients treated with MFAT, they failed to demonstrate significance in their kinetics gait analysis. In contrast, in the present study, statistical significance was obtained exclusively by dividing the investigation by the affected joints [81]. Similarly, patient size variabilities, such as the different joints examined, may have contributed to the synovial fluid assessment and cytokine analysis, which was often insufficient for the entire examination.

The absence of complications related to the intra-articular use of MFAT, despite the small sample size, is undoubtedly a promising result and in harmony with the literature [34,51,52,53,54,55,56]. Furthermore, Zeira et al. showed very high feasibility and safety in the context of these therapies on a much more extensive sample, using intra-articular MFAT in 130 dogs affected by OA [34]. Nonetheless, further studies are needed to determine both the regenerative properties and the maximum duration of positive effects. Furthermore, the actual regenerative capacity of MFATs, although demonstrated in vitro, has never been proven in vivo [95,96,97,98].

Although some authors suggest poor correlations concerning inflammatory biomarker concentration and clinical assessments [92,93,94], in our study, the results obtained from the cytological examination and principal inflammatory cytokines concentration in the synovial fluid can provide important insights, corroborating the clinical outcome of MFAT treatment.

Additional studies could be undertaken with this focus, for example on proteomics, to understand which regulatory activities these cellular fragments have in an inflammatory environment; the cultural characteristics of the synovium cells that could be used as intra-articular therapy [99,100,101,102,103,104,105,106]; and even the interaction of intra-articular MFAT treatments with certain molecules, including nerve growth factor (NGF), which is known as an important pronociceptive factor [107,108,109,110]. This future research could better explain the pain-relieving effect of SVF and MFAT found in the literature [107,108,109,110].

## 5. Conclusions

To the best of our knowledge, this is the first prospective controlled in vivo study which evaluated the efficacy of autologous MFAT in the treatment of OA in dogs. Our results revealed that the use of intra-articular MFAT in dogs is safe and feasible with a single injection. Moreover, it demonstrated superior clinical efficacy compared to the control group in both short- and long-term outcomes concerning OA-derived pain and lameness, considerably reducing intra-articular inflammation. This therapy can be considered a valid and effective therapeutic option for canine OA and hopefully, in the future, can be translated into human medicine.

## Figures and Tables

**Table 1:** Summary and timing of the clinical trial procedures.

Trial Procedure	T0 (Start Trial)	T1 (30 Days)	T2 (60 Days)	T3 (180 Days)
Canine Brief Pain Inventory	X	X	X	X
Specialistic clinician assessment	X	X	X	X
Radiographic examination	X	X	X	
Synovial fluid examination	X	X	X	
Synovial Assay Test	X	X	X	
Intra-articular injection control group	X			
Intra-articular injection study group	X			

**Table 2:** Modified Lameness grading System.

Walk/Trot	Clinical Signs
I	No lameness noted at a work or trot
II	No lameness at a walk, mild lameness at a trot
III	Mild lameness at a walk, significant lameness at a trot
IV	Significant lameness at a walk, non-weight-bearing at trot
V	Non-weight-bearing lameness at a walk and a trot
Standing	
I II III IV V	Normal weight-bearing at a stance Mild decrease in weight-bearing at a stance Significant decrease in weight-bearing at a stance Occasional toe-touching at a stance Holds limb off the ground at a stance
Contralateral LIMB	
I II III IV V	Readily accepts contralateral limb being held up and bears full weight on affected limb Offers resistance to elevation of contralateral limb but bears full weight on affected limb for more than 1 minute after contralateral limb is elevated Offers moderate resistance to elevation of contralateral limb and replaces it after 30 s Offers resistance to elevation of contralateral limb and replaces it after 10 s Refuses to raise contralateral limb
Pain on Palpation	
I II III IV V	No sign of pain during palpation of affected joint/bone Sign of mild pain during palpation of affected joint/bone; dog turns head in recognition Sign of moderate pain during palpation of affected joint/bone; dog pulls limb away Sign of severe pain during palpation of affected joint/bone; dog vocalizes or becomes aggressive Dog will not allow examiner to palpate joint

**Table 3:** Radiographic modified scale of osteoarthritis.

Radiographic Sign	0	1	2	3	4
Osteophytes	Absence	<1 mm	1–2 mm	2–3 mm	>3 mm
Bone sclerosis	Absence	Localized	Pervasive		
Joint narrowing and/or incongruence	Absence	Mild <25%	Moderate 25–50%	Serious >50%	Joint deformity
Capsular ectasia	Absence	Evident			
Final Score	0	1–3	4–6	7–9	>10
Oa Grade	0	1	2	3	4

**Table 4:** Cytological score for synovial fluid evaluation in dogs with OA.

Cytologic Sign		1	2	3
Inflammatory cells		Absence	Average prevalence	High prevalence
Synovial cells		Absence	Average prevalence	High prevalence
Cartilage fragments		Absence	Average prevalence	High prevalence
Blood contamination		Absence	Average prevalence	High prevalence
Matrix		Absence	Average prevalence	High prevalence
Final Score	1–2	3–5	6–9	>10
Synovial Score of OA	1 Paraphysiological synovial fluid	2 Mild inflammation of the synovial fluid	3 Medium inflammation of the synovial fluid	4 Severe inflammation of the synovial fluid

**Table 5:** List of enrolled patients; complete signalment data and randomization. (F = female; M = male; M/N = neutered male; F/OE = neutered female; A = MFAT group; B = HA group).

Patient	QuickCalcks (Random Generator)	Bread	Weight (kg)	Sex	Age	BCS	Joint Affected by OA
1	A	Mixed breed	17	F/OE	7	7	Left shoulder
2	B	Pit bull	27	M	4	5	Right shoulder
3	A	Mixed breed	21	F/OE	6	8	Right shoulder
4	B	Mixed breed	18	M/N	13	6	Left elbow
5	A	Armstaff	25	F/OE	6	5	Left stifle
6	B	Maremma shepherd	47	M	6	7	Right tarsus
7	A	Mixed breed	31	F/OE	5	8	Right stifle
8	B	German sheperd	32	F	10	5	Left elbow
9	A	Labrador retriever	41	F	7	8	Right shoulder
10	B	Mixed breed	26	M	11	3	Left stifle
11	A	Mixed breed	10	M	12	8	Right hip
12	B	Labrador retriever	39	M	12	8	Right shoulder
13	B	Mixed breed	15	F/OE	10	5	Left stifle
14	B	Golden retriever	36	F/OE	3	6	Left hip
15	A	Mixed breed	12	F	7	8	Left hip
16	B	Golden retriever	40	M	5	7	Right hip
17	B	Labrador retriever	36	F	8	8	Right shoulder
18	B	Mixed breed	27	F	7	6	Right stifle
19	A	Chow-Chow	31	F/OE	9	8	Right elbow
20	A	Samoyed	37	M	10	8	Left elbow
21	B	Mixed breed	31	M	10	6	Right stifle
22	A	Labrador retriever	41	M/N	12	8	Left stifle
23	B	Parson terrier	9	F/OE	7	7	Left stifle
24	B	Golden retriever	27	F	5	5	Right elbow
25	A	Labrador retriever	38	F/OE	5	6	Left elbow
26	A	Mixed breed	33	M	6	6	Left elbow
27	A	Fox terrier	10	M	8	6	Right shoulder
28	B	Labrador retriever	25	M	7	3	Right elbow
29	B	Mixed breed	35	F/OE	11	3	Right shoulder
30	A	Duchshound	7	F/OE	8	6	Left shoulder
31	A	Mixed breed	31	F	9	3	Right elbow
32	A	Mixed breed	35	M	7	3	Left elbow
33	B	Boxer	27	F	8	3	Left hip
34	B	Australian shepherd	18	M/N	7	3	Right shoulder
35	A	Dalmatian	23	F	5	3	Right shoulder
36	A	German shepherd	24	F/OE	9	2	Right hip
37	B	Maremma shepherd	42	M	8	5	Left shoulder
38	A	Border collie	15	F	4	2	Left hip
39	B	Mixed breed	25	F/OE	8	3	Left stifle
40	A	Mixed breed	40	F/OE	8	3	Right shoulder

## Data Availability

The clinical data used to support the findings of this study are included within the article.
